# Effect of motor imagery and actual practice on learning professional medical skills

**DOI:** 10.1186/s12909-020-02424-7

**Published:** 2021-01-18

**Authors:** Christian Collet, Mahmoud El Hajj, Rawad Chaker, Bernard Bui-Xuan, Jean-Jacques Lehot, Nady Hoyek

**Affiliations:** 1 Inter-university Laboratory of Human Motor Performance (LIBM - EA 7424), Université de Lyon, Université Claude Bernard, Lyon 1, 27 & 29 Boulevard du 11 Novembre 1918, F-69622 Villeurbanne Cedex, France; 2 ISPEF, Laboratory of Education Cultures and Politics, University Lumière Lyon 2, Lyon, Cedex 07, 69365 France; 3 Health Services and Performance Research (HESPER - EA7425), Faculty of Medicine Lyon-Est, Université de Lyon, Université Claude Bernard Lyon 1, 8, Avenue Rockefeller, F-69008 Lyon, France; 4 Hospices Civils de Lyon, Neurological Hospital, Boulevard Pinel, 69 500 Lyon, Bron France

**Keywords:** Motor imagery, Motor learning, Professional medical skills

## Abstract

**Background:**

The peripheral venous catheter is the most frequently used medical device in hospital care to administer intravenous treatment or to take blood samples by introducing a catheter into a vein. The aim of this study was to examine the effect of motor imagery associated with actual training on the learning of peripheral venous catheter insertion into a simulated venous system.

**Method:**

This was a prospective monocentre study in 3rd year medical students. Forty medical students were assigned to the experimental group (*n* = 20) performing both real practice and motor imagery of peripheral venous catheter insertion or to the control group (*n* = 20) trained through real practice only. We also recruited a reference group of 20 professional nurses defining the benchmark for a target performance.

**Results:**

The experimental group learned the peripheral venous catheter insertion faster than the control group in the beginning of learning phase (*p* < 0.001), reaching the expected level after 4 sessions (*p* = .87) whereas the control group needed 5 sessions to reach the same level (*p* = .88). Both groups were at the same level at the end of the scheduled training.

**Conclusions:**

Therefore, motor imagery improved professional motor skills learning, and limited the time needed to reach the expected level. Motor imagery may strengthen technical medical skill learning.

## Background

The insertion of peripheral venous catheter (PVC) is one of the most common medical procedures used by clinicians. It involves inserting a catheter into a vein to administer intravenous therapy or to allow blood collection. There are potential septic risks for the patient [1], thus, learning how to insert a PVC reduces potential traumatic or infectious lesions and limits the risk of errors [2–4]. Medical techniques are learnt through training procedures. However, due to the short time allocated, and the amount of students involved, the training time is often limited. Mental simulation may provide alternative methods [5]. With respect to the principle: *“never the first time on the patient”* [6], simulation may offer a reliable framework without any risk for the patient (practiced on a manikin). Motor imagery (MI) may be associated with actual execution during training sessions [7–12], among other methods (e.g. observation, virtual reality). Motor imagery is a top-down process mainly based on recalling the sensory information usually generated by actual execution [4, 13]. We have the ability to self-represent our own actions through their main visual features (shapes, outlines, colour and movements) and bodily-evoked sensations (tactile or kinesthetic).

When associated with actual movement, MI is believed to enhance motor skills learning, and to facilitate the recover of motor ability after central or peripheral injury [4, 5, 14–16]. There is also strong evidences that MI may help controlling our own activation level through specific mental representation likely to increase or decrease the general arousal of the organism. [5]. Several studies highlighted the positive effects of MI [17–19], particularly in surgery and motor rehabilitation where the cognitive demand is high [10, 11, 20–27]. Experienced surgeons view MI as the most effective procedure for complex and stressful situations preparation, probably because the cognitive demands are close to those mobilized during MI, e.g. the ability to self-represent the spatial environment in which surgery will take place [9, 13, 26, 28]. Planning the needed actions before their execution, memorizing mental references and transferring mental abilities for real practice are the main operations that can be supported by MI [14, 25–30]. Associating MI to real practice may thus favour the learning in health professions [7].

The extent to which MI can complete or even compensate and substitute for the effect of actual practice should be better investigated as well as its potential effects on learning medico-surgical techniques under limited practice time. The main objective of this study was to assess whether MI associated with actual training improved PVC insertion.

## Methods

### Experimental design

The experiment included a pretest (session 1), 4 learning sessions and a post-test (session 6). During the first session, the students watched an instructional video on PVC insertion, then performed the technique twice under the supervision of 2 trainers that were naive in relation to the objectives of the experiment. Each student inserted the PVC on a double skin placed on the forearm of another student, who played the role of a patient. There was however no patients actually requiring the pose of a PVC. The double skin was an elastomeric venous system into which simulated blood was introduced through a syringe. The experimenters evaluated the quality of the PVC insertion through the respect of each stage using a common rating scale where they indicated whether the participants respected the aforementioned steps or not. We also recorded the timing of the PVC insertion.

**Fig. 1:**
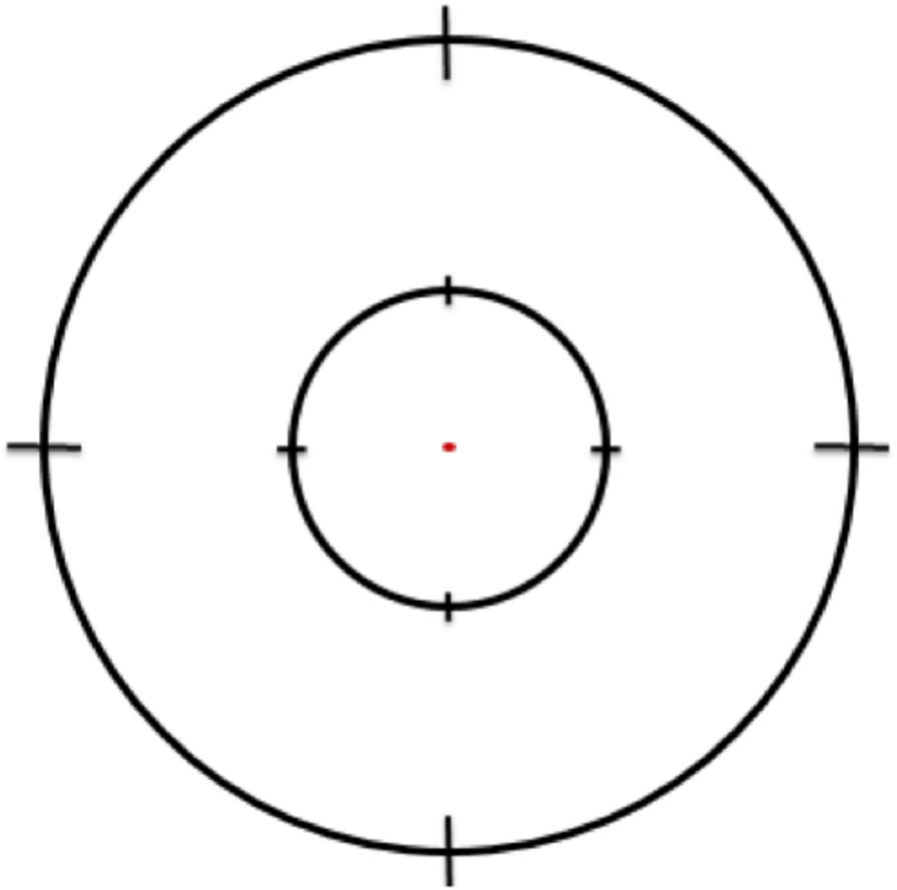
Test of MI imagery quality through mental chronometry

**Fig. 2:**
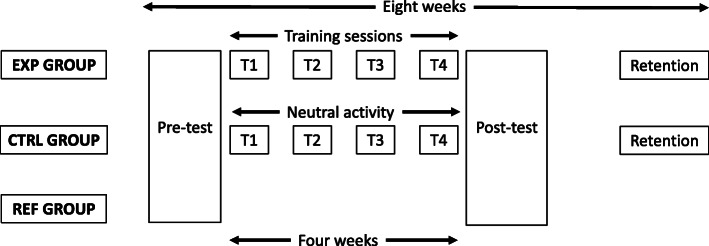
Summary of the experimental design, including the experimental group (exp), the control group (ctrl) and the reference group (ref)

At the end of the first session, we evaluated the students MI ability with the Motor Imagery Questionnaire (MIQ-3) [31], and a mental chronometry test (Fig. 2). We used the most recent version of the Movement Imagery Questionnaire (MIQ-3), early proposed by Hall and Pongrac (1983) [32] and later revised by Hall and Martin (1997) [33]. This test consists of 12 elements describing real motor situations to be mentally reproduced. The participants assessed each item through a 7-level Likert scale (maximal score = 84 points) and then performed the mental chronometry test on a A4 printed sheet. The test requested to point towards 8 targets placed on two circles in a predetermined order, at free self-pace. The first 4 pointing towards the targets of the small circle were clockwise, and the next four, on the large circle, were counterclockwise. The participants started from the center of the circles and went back to this position, after pointing towards each target (Fig. 1). Both served as landmarks for each participant to start and stop the timer during MI. We recorded the timing of the 8 pointing during actual execution. Then, each participant mentally performed the same task. The comparison of real and imagined timing was an index of MI quality. The more MI duration matched actual duration (i.e. isochrony), the better the quality of MI, relative to speed preservation. As both the MIQ-3 and pointing towards targets tests addressed different components of MI quality, Williams et al. (2015) advised to use them together for a more comprehensive assessment of motor imagery ability [34].

### Patient and public involvement

The participants were forty third-year medical students (23 women, 17 men, mean aged 20.6 ± 1.0) at Claude Bernard University Lyon 1, France. Twenty professional nurses (aged 40.4 ± 4.0) formed the reference group (ref). All participants signed an informed-consent form before the study. The management and ethics committee of the Faculty of Medicine (Lyon-Est) approved the experimental design after the experimenters presented the aims and scopes to the scientific board council. The study also obtained the scientific support of the Center for Education through Simulation in Health (CLESS), hosted by Lyon1 University. We divided the 40 students into two homogeneous groups, the experimental (exp - 13 women and 7 men, mean age = 20.5, ± 1) and the control (ctrl - 10 women and 10 men, mean age = 20.7 ± 0.7), according to their gender, MI abilities and early PVC insertion performance PVC insertion. The exp group trained with a mixed schedule associating actual practice (AR) and motor imagery (MI). The ctrl group performed two neutral tasks for equivalent duration as to the exp group (looking at videos about medical care and reading articles related to ethics and palliative care). Although linked with clinical care, the neutral task avoided any relation with MI. The exp and the ctrl groups trained during 4 successive sessions after the pre-test. Then, the participants performed the post-test and the retention test 1 month later. The overall design was spread over 8 weeks to distribute the learning sequences across time. The ref group only performed the pre-test and the post-test (See Fig. 2 and Table 1).

**Table 1:** Summary of the experimental design

Content	Session Duration	EXP (MI) Group	Session Duration	CTRL Group
Session 1 (Pretest)	2 h	MIQ-3 & mental chronometry – Practical discovery of the clinical technique. Video instruction - observation + actual repetition of the skill under the same conditions: instructor and script). Evaluation: timing of the last AP		
Session 2	1 h	[AP/MI/AP]*3 + [MI/AP/MI] Evaluations + timing the 2nd and the last AP MC + Quality of the 5th MI	1 h	[AP/N/AP]*3 + [N/AP/N] Evaluations + timing the 2nd and the last AP
Session 3	1 h	[AP/MI/AP]*2 + [MI/AP/MI]*2 Evaluations + timing the 2nd AP MC + Quality of the 5th MI Timing the AP just after last MI	1 h	[AP/N/AP]*2 + [N/AP/N]*2 Evaluations + timing the 2nd and the last AP
Session 4	1 h	[AP/MI/AP] + [MI/AP/MI]*3 Evaluations + timing the 2nd AP MC + Quality of the 6th MI Timing the AP just after last MI	1 h	[AP/N/AP] + [N/AP/N]*3 Evaluations + timing the 2nd and the last AP
Session 5	1 h		1 h	
Session 6 (Post-test)	1 h		1 h	
Retention	1 h	Assessments – timing of actual execution		

*MC* Mental chronometry *AP* Actual practice, *MI* Motor imagery, *N* Neutral task, *x = Number of repetitions of the sequence

We requested the exp group to alternatively perform actual practice and MI according to the experimental design, i.e. increasing the rate of MI trials across sessions, as showed in Table 1. The exp group performed 36 trials (18 actual and 18 MI).

We read the MI script once for the first MI trial when starting each session as follow: i) Isolate yourself from the environment by closing your eyes and disregard the possible sound environment; ii) Represent all steps of the PVC insertion as an actor, i.e. as if you had to perform the movement yourself; iii) Use sensory information as a support for the construction of MI, mainly tactile (contact with patient’s skin and all materials), visual (vein tracking, angle control between the needle and the skin) and proprioceptive (muscle effort, joint position, resistance offered by the skin to the needle insertion ...); iv) Stay motionless during MI, without mimicking the action; v) Start the sequence when spreading and stretching the skin; vi) Stop the action when you place the mandrel into the needle collector.

The MI script described the main steps of the sequence, with the key information for a skilled execution. We thus gave several instructions as follow: *“When you represent the action, visualize all movements as parts of the action. To do this, close your eyes and try to clearly perceive all the steps of the PVC insertion, as if you were actually performing the movement. You should not move or mimic the movement. Place yourself in relation to the patient, as if you were going to actually make the needle insertion”*.

1. Spread and stretch the skin with your left hand. Locate the exact place where you need to insert the needle.
2. Check the needle-skin angle before insertion.
3. Insert the needle into the vein by exerting the proper force, try to perceive the resistance feedback of the tissues the needle crosses.
4. When in the vein lumen, stabilize the needle.
5. Insert the catheter, bevel up. Check that the angle is correct, apply the appropriate force, perceive the resistance that the tissues oppose to you.
6. Force the passage of the catheter into the vein lumen.
7. Monitor the arrival of blood in the mandrel.
8. Slowly slide the catheter over the needle to position it in the vein.
9. Loosen the tourniquet with one hand and hold the catheter with the other.
10. Place the mandrel into the needle collector.

Since PVC insertion is a complex procedure, we determined two accurate boundaries corresponding to the first step (“spreading and stretching the skin …” ) and the tenth and last step (“placing the mandrel into the needle collector”). The boundaries were determined through the professional analysis of the nurses responsible for student training, starting and ending the main 10 sequences constituting a usual PVC insertion. It was important to determine precise benchmarks so that the timing of both actual execution and MI was carried out on clear and precise indices. We therefore studied a specific part of the whole PVC insertion so that the MI script can be easily understood and memorized, with both accurate timing during actual execution and MI.

### Dependent variables

The *duration of actual PVC insertion* was an index of skilled action. We thus timed the last actual trial of each participant, at the end of each session in the exp and ctrl groups. We also timed *MI accuracy* from sessions 2 to 5, by comparing actual duration to that of the imagined action. The exp group triggered the timer when starting the MI sequence and stopped it at the end. The exp group also assessed the *vividness* of MI on a 7-level Likert scale, from very blurred image (level 1) to a ultra-high definition image (level 7), intermediate levels being used for intermediate vividness. This was just a self-assessment, designed to check that MI was of enough high quality and consider that MI training was reliable.

Two expert instructors evaluated the *quality of the PVC insertion* in the exp and the ctrl groups. They were naive with respect to the objectives of the study and uninformed of the participants’ home group. They used a specific evaluation grid from the CLESS pedagogical team, made of 5 items that should be considered “reached” or “not reached” and completed at the end of each session. We assessed the quality of the PVC insertion during the last physical trial of each session, and gave a feedback to each participant to help them better memorizing the procedure. This grid only served to guide the participants with a formative objective and was not included into the statistical design.

The actual duration was the dependent variable in the ref group. We timed a real trial to be compared with that of the exp and ctrl groups.

The participants completed the retention test 1 month after the post-test (Fig. 2). Each participant only performed one real trial and, additionnaly, those from the exp group performed one MI trial. We evaluated the quality of the PVC insertion similarly as during previous tests (actual and MI duration).

### Statistical analysis

We processed data of the last real trial of each session with a linear mixed model analysis. We thus integrated group and learning sessions (and their interaction) as fixed-effects, and a random subject effect, with the PVC insertion time as the dependent variable. We thus aimed at comparing performance evolution along the learning process, i.e. among the pretest, the four learning sessions, the post-test and the retention test in each of the 3 groups (exp, ctrl and ref). We also aimed at comparing the actual duration of PVC to that of MI in the exp group. Finally, the ref group represented the expected level to reach at the end of the learning process and only performed the pre- and the post-test. We set the statistical threshold for significance at .05.

## Results

### Descriptive data

We used a 1 to 7 Likert scale with the aim to assess and control MI vividness, i.e. the quality of training sessions. The self-evaluation of MI vividness by the participants of the exp group always remained above the median value (4). Mean values (SD) were 5 (0.86), 5.50 (0.69), 5.65 (0.67) and 5.58 (0.69) from the first to the fourth training session.

During the pretest, the average duration of PVC insertion was 23.8 ± 4.8 s in the exp group, 23.3 ± 5.1 s in the ctrl group and 12.4 ± 2.7 s in the ref group. The linear mixed model analysis showed a significant group effects, F (2, 313.80) = 54.45; *p* < .001. There was also a significant session effect, F (6, 117.95) = 44.55; *p* < .001 and of the interaction group*session, F (12, 117.75) = 12.02; *p* < .001. The ref group obviously outperformed the two others after the pre-test, mean differences being 10.90s in the ctrl group and 11.44 s in the exp group (see Table 2).

**Table 2:** Detail of the linear mixed-effects model with PVC insertion time as a dependent variable

Session	Group	Estimate	Std. Error	df	t	*P*
*Intercept*	REF	12.4	.61	78.04	20.10	.001
Pre-Test	CTRL	10.9	1.10	121.08	9.89	.001
	EXP	11.4	1.10	121.08	10.38	<.001
Session 2	CTRL	9.6	1.20	109.74	7.95	.001
	EXP	5.2	1.20	109.70	4.33	.001
Session 3	CTRL	5.5	.88	106.78	6.24	.001
	EXP	3.1	.88	106.78	3.53	.001
Session 4	CTRL	3.5	.84	94.47	4.06	.001
	EXP	0.5	.84	94.47	.53	.59
Session 5	CTRL	−0.2	.84	90.76	−.15	.88
	EXP	−0.5	.84	92.01	−.55	.58
Post-Test	CTRL	−0.4	.86	93.35	−.47	.64
	EXP	−2.0	.86	93.35	−2.28	.13
Retention test	CTRL	−0.3	.85	78.04	−.34	.73
	EXP	−1.6	.85	78.04	−1.85	.07

More interestingly, when taking the ref group as a constant of the model (intercept, M = 12.4 and SE = 0.61), results by group and session showed significant interactions between the 3 groups until the fourth session. We first checked that the performance of the ref group remained constant between the pre and post-test (t = 0.54, *p* = .96, NS, mean duration for insereting the PVC being 12.4 s ± 2.7 and 12.3 s ± 2.2 during the pre and the post test, respectively). The exp group reached the level of the ref group at session 4 whereas the ctrl group needed to attend the fifth session to reach the same level. There was still a difference between the ref and the ctrl group after the fourth session (t = 4.06, *p* = .001) whereas no difference emerged between the exp and the ref groups (t = 0.53, *p* = .59), mean difference being 3.5 s and 0.5 s, respectively (see Table 2, session 4). The mean difference between the ref and the ctrl groups did not reached significance after the fifth session (t = − 0.15, *p* = .88).

**Fig. 3:**
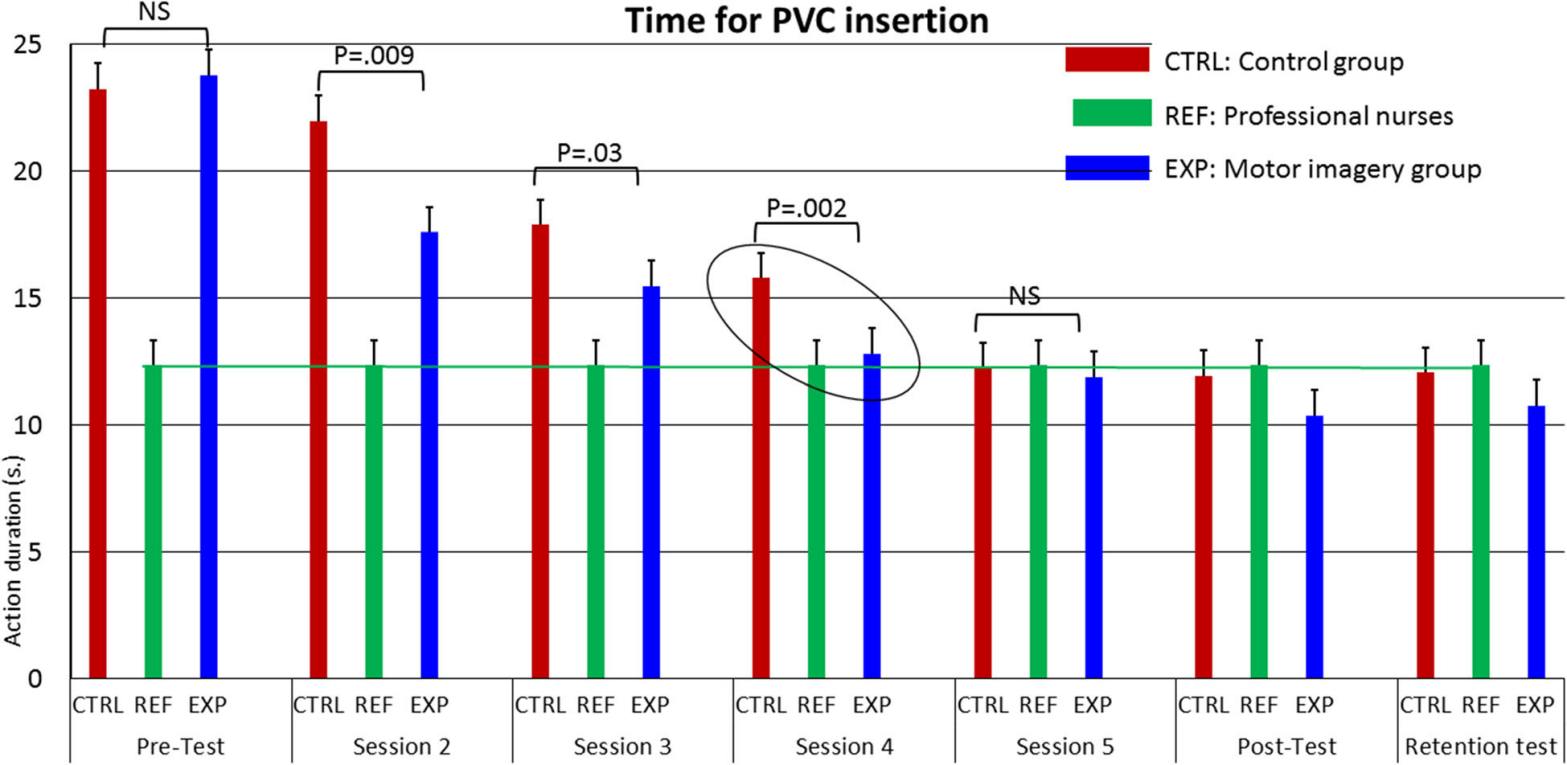
Comparison of the PVC duration according to groups and sessions. The values are expressed by the difference between the mean of the ref group and those of the exp and the ctrl group. Duration decreased in the exp and ctrl groups along the training sessions but the decrease is greater in the exp group who trained both with actual execution and MI. The ellipse highlights on the main data of the experiment: It only takes 4 training sessions to reach the target performance of the ref group while the CTRL group needed 5 sessions to achieve this goal

The comparison of the exp to the ctrl group did not show any difference after the pre-test. The average duration of PVC insertion was 23.8 ± 4.8 s and 23.3 ± 5.1 s, respectively. We observed significant differences between the same two groups after sessions 2, 3 and 4, the exp group systematically displaying faster PVC insertion time than the ctrl group. After session 2, t = − 2.75; *p* = .009; d = .87, mean duration being 17.6 ± 4.7 s and 21.9 ± 5.31 s, respectively. After session 3, t = 2.31; *p* = .03; d = .73, mean duration being 15.5 ± 3.1 s and 17.9 ± 2.1 s, respectively. This difference remained after session 4, t = 3.41; *p* = .002; d = 1.07, mean duration being 12.81 ± 2.1 s and 15.8 ± 3.3 s, respectively. No significant difference emerged between the two groups after the fifth session, the post-test and the retention test, as summarized by Table 2 and Fig. 3.

### Comparaison of actual duration (AD) to MI duration of (MI_D) in the exp. group

**Fig. 4:**
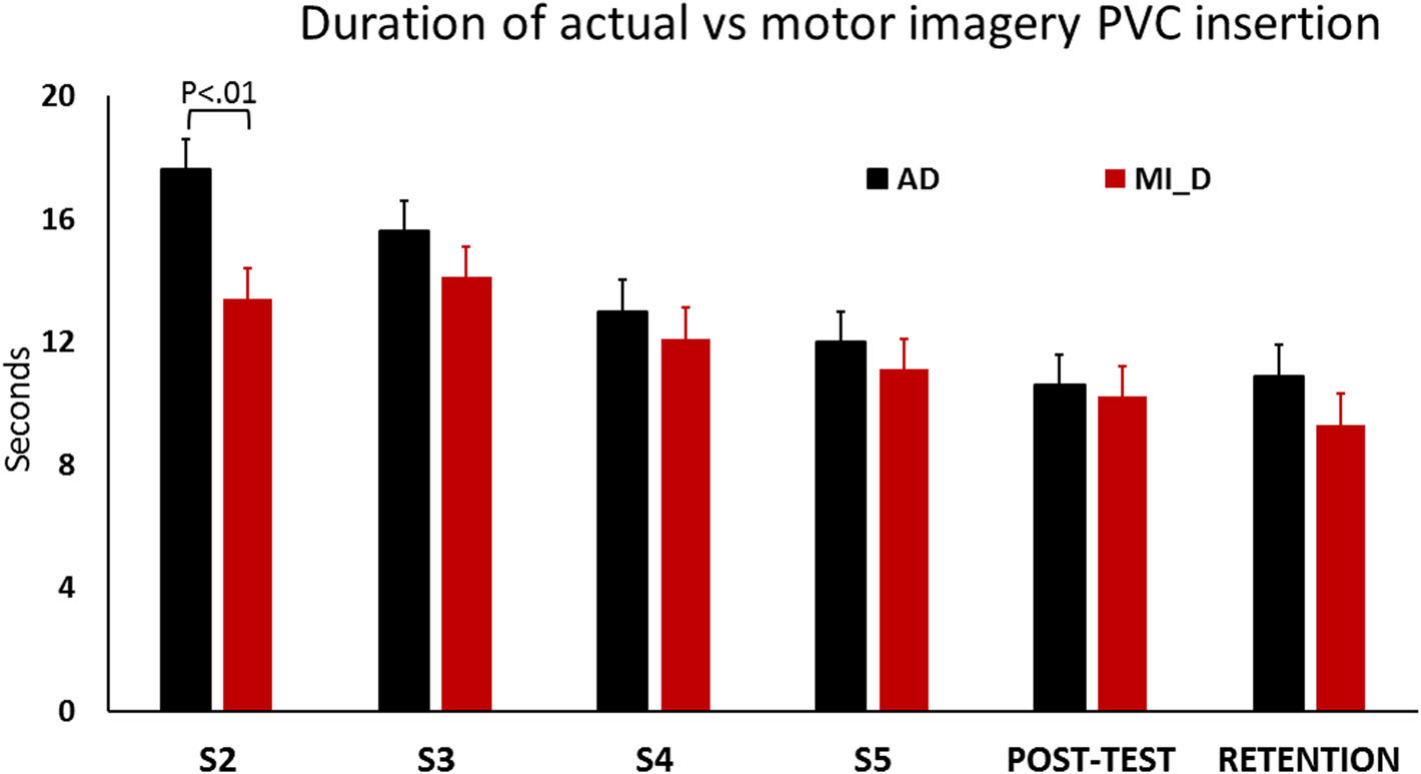
Comparaison of actual duration (AD) to MI duration (MI_D) needed to insert a PVC, in the exp. group, during the 4 training sessions (from S2 to S5), the post-test and the retention test

Another interesting issue was to compare the evolution of the actual durations of PVC insertion under actual (AD) and imagined conditions (MI_D). The linear mixed model analysis comparing actual duration to MI duration showed a marginally significant difference, F (6, 117.95) = 3.54, *p* = .07. The t-test confirmed that only session 2 showed differences between actual and MI duration, mean (SD) being 17.6 (±4.7) and 13.3 (±4.3), respectively (see Fig. 4). The effect size based on Cohen’s d was 0.35, showing a main effect between low and median.

## Discussion

The main objective of the experiment was to study the effect of Motor imagery (MI) associated with actual practice on the ability to learn how to insert a PVC. We hypothesized that MI should improve learning and make the skills more effective after a four-session training period [3, 7, 16]. We first checked that the MI training sessions were adequately performed by assessing the MI vividness. The Likert scores were all above the median values and thus attested that no participant from the exp group encountered difficulties in representing the different sequences of the PVC insertion.

We then tested the quality and the speed of PVC insertion, two factors attesting that performance reached this goal. The exp group placed the PVC faster than the ctrl group as early as the second session. The quality of PVC placement was assessed by blind experts and was comparable in both groups during the training sessions and the retention test. Therefore, MI training only improved the speed, but not the quality of the skill. We should nevertheless mitigate this conclusion because the evaluation grid was not enough discriminating. It was made of 5 items, summarizing the main objectives to be achieved to insert a PVC, which were simply assessed as ‘achieved’ or ‘not achieved’. A more precise assessment tool should be developed in order to monitor the ‘insertion quality’ factor as accuratetly as the ‘speed’ factor.

The exp group reached the expected level after 4 sessions, and was faster than the ctrl group from session 2 to 4. This was probably due to the association between MI training and actual practice since we observed isochrony from the third session while the quality of MI remained at the same level. A gain in speed usually causes a decrease in accuracy, as described by Fitts law (1964), and we could have observed an improvement in speed at the expense of execution quality [35]. The speed was improved but not in conjunction with a decrease in accuracy. The participants may have given priority to accuracy as this is the first requirement of the PVC insertion. It is a fine and complex skill which must preserve the patient’s body integrity. There is a trade-off in favour of accuracy at the expense of speed. As previously observed by Decety and Jeannerod (1995), Fitts law also applies in motor imagery [36]. Secondly, the experiment did not include temporal constraints. Time pressure is worthy to be included into the protocol as it is a frequent factor in clinical practice. We did not test this variable as the participants were novice in PVC insertion and workload must be controlled at the start of learning. Despite this experimental condition, we observed a tendency to insert the PVC faster after the ref duration was reached. The next goal of learning in novice people will be to strengthen the accuracy of PVC insertion before doing it more quickly, the speed being here a secondary objective compared to the accuracy. In more experienced people, automation would be the response to time pressure, high speed constraints and accuracy requirement. Indeed, automated skills are highly resistant to any external (time pressure, distractor) or internal disturbance (emotional load). Under experimental conditions where time constraints were high, De Witte et al. (2018) nevertheless observed a positive effect of MI training on the quality of suturing and knot tying by novice surgeons [11]. The first stages of motor learning are thus conducive to progress, both in speed and execution accuracy.

Conversely, Jungmann et al. (2011) did not show an effect of MI on learning procedures in surgical novices who completed additional mental practice during the interval among actual training sessions [37]. In massed learning procedure, training is concentrated over a short period of time and is less effective than when the learning sessions are distributed over time [11], especially for novice practitioners. The distributed procedure also brings more marked progress when the technique to learn is complex. Neuronal plasticity is better stimulated as this process requires time to operate, especially in novice learners [38]. Thus, the conditions of MI practice strongly influence the progress made.

The exp group performed significantly better than the ctrl group as early as the second training session showing that MI is quickly effective. This effect still persisted during the following sessions with faster insertion of PVC in favour of the exp group during both sessions 3 and 4. Then, the effects of MI subsided slightly, with the ctrl group performing as well as the exp group. During initial learning where a high volume of knowledge was to be learnt [27], MI better concretized the operations to be carried out and made possible to segment them into several sub-objectives. The association of MI with real practice led to better efficiency than in the ctrl group, the efficiency being defined as the ability to achieve the best result with minimum resources. In our experiment, the learning time was reduced thank to MI and represented a saving of resources, particularly during the first steps of learning. Although the two groups achieved comparable final performance, learning was nevertheless facilitated during the early phases, as previously shown by Guillot et al., (2009) [39]. Mulla et al. (2012) observed that mental training alone cannot replace conventional training [40]. However, MI is closely involved in memory processes and enriches the cognitive phase of learning. This may explain that the exp group improved faster than the ctrl group at the early stages of learning. The association of MI with real practice accelerated motor skills learning as the exp group reached the expected level during the 4th session, whereas 5 sessions were needed in the ctrl group, despite no strong difference in performance were observed from the 3rd session.

Mental chronometry also highlighted that the duration of the mental representation slightly underestimated that of real execution, at the beginning of the second session (although this was a marginally significant difference). The second session was the first with MI and probably required habituation. Underestimation may also be due to task complexity, the mental representation of complex skills being more difficult than that of simple skills [41]. In general, the beginner showed a tendency to omit key points, thus decreasing mental representation duration [27]. As shown by the low/median effect size, the difference was relatively weak and isochrony was found during session 3 and 4. Isochrony explains the positive effect of MI since MI mentally reproduced the physical constraints of the task and thus benefited the actual execution. Motor imagery and actual practice reinforce each other during training and isochrony is therefore an indicator of the quality of mental work. During the third session, the duration of actual practice decreased due to skill execution improvement while that of MI increased, thus attesting that the exp group took into account most elements of the script and that the quality of mental representation improved. This resulted in better congruence between actual and imagined execution times, close to isochrony, thus confirming that both tasks share similar processes. In particular, the programming phase prior to actual execution shares a set of operations with the mental representation of the movement [17, 18]. MI improved the learning speed of PVC insertion and influenced actual practice [19, 20]. In turn, actual practice also modulated MI, both reinforcing each other. Thus, the decrease in the duration of the actual practice resulted in a decrease in MI duration.

The main perspective that could be addressed would be to study the role of MI in more complex conditions of PVC insertion, e.g. in patients with a fragile venous network, or under time pressure and stressful conditions, where there is an urgent need to pose a PVC. More generally, defining more precise and operational criteria of PVC insertion through the Objective Skills Assessment Tool (OSAT), could complement the evaluation of PVC training [42].

## Conclusion

The main strength of this study is the main positive effect of MI on movement duration, in association with actual practice. Professional motor skills can be learnt and improved through their mental representation especially during the early stage of learning. Motor imagery provides a complementary procedure that accelerates learning. This is a crucial point in the medical professions where the duration of practice is often reduced. If MI is carried out with respect to its rules of practice, it can have a satisfactory pedagogical effect but also an economical impact by reducing the training costs (number of sessions with time and device gains) while improving the quality of learning.

The limits of our results are that the effects of MI appear to be rather weak and limited to the initial phase of learning. However, other factors could be included in a complementary future study, such as the individual capacities of MI, for example.

As a result, the quality of care provided to patients should also be improved. The collaboration between medicine/surgery and cognitive neuroscience research thus needs to be strengthened with the aim to improve motor learning in the field of medico-surgical techniques.

## Data Availability

The datasets collected, used and analysed during the current study are available from the corresponding author on reasonable request.

## Abbreviations

AD: Actual duration
AP: Actual practice
CLESS: Center for education through simulation in health
CTRL: Control group
EXP: Experimental group
MC: Mental chronometry
MI: Motor imagery
MI_D: Motor imagery duration
MIQ-3: Motor imagery questionnaire
N: Neutral task
PVC: Peripheral venous catheter
REF: Reference group
*x: Number of repetitions of the sequence
